# Job Satisfaction and Work-Related Quality of Life Among School and Clinical Nurses: A Cross-Sectional Study

**DOI:** 10.3390/healthcare14050604

**Published:** 2026-02-27

**Authors:** Sokratis Lialias, Vissarion Bakalis, Ioanna Dimitriadou, Maria Saridi, Aikaterini Toska, Ioanna V. Papathanasiou, Pavlos Sarafis, Evangelos C. Fradelos

**Affiliations:** 1 Post-Graduate Program of Primary Health Care, University of Thessaly, 41500 Larissa, Greece; swkratislialias@gmail.com; 2 Department of Nursing, University of Thessaly, 41500 Larissa, Greece; bissarion_bakalis@yahoo.gr (V.B.); ioadimitriadou@uth.gr (I.D.); msaridi@uth.gr (M.S.); ktoska@uth.gr (A.T.); iopapathanasiou@uth.gr (I.V.P.); psarafis@uth.gr (P.S.)

**Keywords:** job satisfaction, work-related quality of life, school nurses, clinical nurses, nursing workforce, turnover intention, working conditions

## Abstract

**Highlights:**

**What are the main findings?**

- School nurses in Greece reported significantly higher job satisfaction and work-related quality of life compared to clinical nurses, while clinical nurses demonstrated a stronger intention to leave their sector.
- Job satisfaction was strongly and positively associated with WRQoL, with working conditions, general well-being, and job–career satisfaction emerging as key predictive factors.

**What are the implications of the main findings?**

- Improvements in organizational and environmental work factors, especially salary, career development opportunities, and working conditions, are essential to strengthen nurse retention and professional well-being.
- Policy strategies targeting workplace quality rather than demographic characteristics may have a greater impact on sustaining the nursing workforce and ensuring healthcare system resilience.

**Abstract:**

**Background/Objectives**: Job satisfaction and work-related quality of life (WRQoL) are essential determinants of nurses’ well-being, performance, and retention. Differences between school and clinical nursing environments may influence these outcomes, yet comparative evidence from Greece remains limited. This study aimed to assess and compare job satisfaction and WRQoL among school and clinical nurses and identify factors associated with professional well-being and turnover intention. **Methods**: A quantitative cross-sectional study was conducted between November 2024 and January 2025 among 165 nurses employed in Greek public hospitals and schools. Data were collected using demographic questions, the Job Satisfaction Survey (JSS), and the Work-Related Quality of Life (WRQoL) scale. Statistical analyses included descriptive statistics, independent group comparisons, correlation analysis, and multiple linear regression. **Results**: Moderate levels of job satisfaction (M = 125.10) and WRQoL (M = 75.27) were observed overall. School nurses reported significantly higher scores in both job satisfaction and WRQoL compared to clinical nurses (*p* < 0.001). Clinical nurses expressed a greater intention to transition to school nursing. Lowest satisfaction levels were related to salary, promotion, and fringe benefits, while supervision, coworkers, and nature of work scored highest. Strong positive correlations were found between job satisfaction and WRQoL dimensions. Regression analysis indicated that general well-being, job and career satisfaction, and working conditions significantly predicted job satisfaction, explaining 54.7% of its variance. **Conclusions**: Professional well-being among nurses is primarily shaped by workplace conditions rather than demographic factors. Interventions focused on improving compensation, career progression, and work environments are critical for enhancing job satisfaction and sustaining the nursing workforce.

## 1. Introduction

Job satisfaction and work-related quality of life are two critical components that can affect the level of healthcare delivery, employee performance and productivity, well-being, job mobility of nursing staff, and their commitment to the organization [1].

Job satisfaction refers to the sense of satisfaction and pleasure an employee derives from his or her job, and to the individual’s general evaluation of his or her occupation as pleasant or unpleasant [2]. According to Locke [3], job satisfaction is the result of the employee’s positive emotional state about his or her occupation or work experiences and the responsiveness of the job to his or her expectations, values and needs [4].

At the same time, working life is an important part of everyday life, and lack of job satisfaction can negatively affect an individual’s perception of quality of life [5]. Work-related quality of life includes work–life balance, a sense of professional success, and avoidance of burnout. Unlike job satisfaction, which primarily reflects an individual’s evaluative attitude toward their job, WRQoL integrates psychological well-being, work–life balance, perceived working conditions, stress, and professional fulfillment. WRQoL therefore captures not only how individuals feel about their job, but also how work influences their general quality of life and functioning [6,7,8].

Although job satisfaction and work-related health quality share a common conceptual background, they represent distinct but interrelated constructs [9,10]. Job satisfaction reflects a cognitive and affective evaluation of job characteristics, while work-related health quality reflects the broader consequences of work on holistic well-being. Existing literature suggests strong associations between these variables, with improvements in WRQoL being associated with improved job satisfaction and reduced job strain [11,12,13,14].

The nursing profession is undergoing a major crisis, with both job satisfaction and work-related health quality taking center stage [15,16]. Studies show that workload, staffing adequacy, professional autonomy, administrative support, working conditions, and occupational stress significantly influence nurses’ professional well-being. Reduced WRQoL has been associated with burnout, emotional exhaustion, reduced job satisfaction, and increased intention to leave [17,18]. Clinical nurses often face high workload, shift-related demands, emotional strain, and increased stress levels, factors that are typically associated with moderate or reduced job satisfaction and quality of work life. In contrast, school nurses often work in environments characterized by more predictable schedules, increased autonomy, and a focus on preventive care. However, challenges such as limited professional development opportunities and limited institutional support can also impact their professional experiences [19].

Despite the growing international interest in nurses’ Work-Related Quality of Life, research specifically focusing on school nurses remains relatively limited, especially in the Greek context. Existing studies in Greece mainly examine clinical settings, often reporting moderate levels of job satisfaction related to financial compensation, workload, and organizational constraints. Comparatively little is known about the professional well-being of school nurses or the potential differences between school and clinical nursing settings [1,16]. Given the structural, organizational, and psychosocial differences between clinical and school nursing roles, comparative research is warranted. Understanding how job satisfaction and Work-Related Quality of Life manifest themselves in these professional groups can provide valuable insights into workforce dynamics, professional well-being, and phenomena related to staff retention.

The present study aims to assess and compare job satisfaction and Work-Related Quality of Life among school and clinical nurses in Greece. Furthermore, the study seeks to identify factors associated with professional well-being and examine the intention to leave within these professional groups.

## 2. Materials and Methods

### 2.1. Study Design

In this study, a quantitative cross-sectional analysis was designed to investigate and determine the levels of job satisfaction and Work-related Quality of Life and their constituent factors among school and clinical nurses working in Greek public schools and hospitals, respectively. The study assessed multiple variables, including demographic characteristics such as age, gender, educational level and years of service, as well as intention to work as either school nurses or clinical nurses. To achieve the study objective, clinical and school nurses worked, respectively, in various public hospitals and primary and secondary schools in Greece. The study was conducted from November 2024 to January 2025, after approval by the ethics committee.

### 2.2. Study Population

The study population consisted of licensed nurses employed in Greek public healthcare and educational settings. Participants were eligible for inclusion if they were actively employed either as clinical nurses in public hospitals or as school nurses in public primary or secondary schools, held a valid nursing license, were able to understand and complete the Greek-language questionnaire, and agreed to participate voluntarily. Nurses employed in private healthcare institutions or private educational settings were excluded to ensure sample homogeneity. Additionally, individuals working exclusively in administrative roles without direct professional practice were not included. Questionnaires with incomplete responses were also excluded from the analysis.

Nurses were invited to participate in the survey via email, social media and professional nurses’ associations. In addition, after being informed, approved and granted permission by the Directorates of Education and the Administrative and Scientific Councils of the hospitals, the link to the online questionnaire was forwarded electronically through the schools and hospitals to the respective nursing staff.

### 2.3. Data Collection

Data were collected using an anonymous, self-administered online questionnaire. The survey link was distributed electronically via email, social media platforms, and professional nursing networks. Following institutional approval, the questionnaire was also forwarded through hospital administrations and school directorates. Data collection took place between November 2024 and January 2025.

The questionnaire consisted of three sections. The first section included demographic and professional characteristics such as age, gender, educational level, years of experience, and turnover intention. The second section incorporated the Greek version of the Job Satisfaction Survey (JSS) [8,20], a multidimensional instrument consisting of 36 items measuring nine dimensions of job satisfaction: pay, promotion, supervision, fringe benefits, contingent rewards, operating conditions, coworkers, nature of work, and communication. Responses are recorded on a six-point Likert scale ranging from strongly disagree to strongly agree. Higher scores indicate greater job satisfaction.

The third section included the Greek version of the Work-Related Quality of Life (WRQoL) scale. The instrument comprises 24 items evaluating six domains: Job and Career Satisfaction (JCS), General Well-Being (GWB), Home–Work Interface (HWI), Control at Work (CAW), Working Conditions (WCS), and Stress at Work (SAW). Items are rated on a Likert scale, with higher scores reflecting better perceived quality of working life, except for the stress dimension, where higher scores indicate greater occupational stress [21,22].

Participation was voluntary, and respondents were informed about the study purpose, confidentiality, and their right to withdraw at any time.

### 2.4. Statistical Analysis

The data obtained from the present study were analyzed with the Statistical Package for Social Sciences (SPSS) software, version 27.0, using descriptive and inferential statistics. The level of statistical significance was set equal to 0.05.

Measures of central tendency and dispersion, coefficient of asymmetry and kurtosis, and Kolmogorov–Smirnov normality test values were calculated for all scales and subscales.

To investigate the statistically significant correlations between the scales of the two research instruments of work-related quality of life and job satisfaction, the calculation of Spearman’s correlation coefficient and Pearson’s coefficient was performed.

To conduct the individual comparisons between the scales of the questionnaire, appropriate normality tests and the conditions for applying parametric tests were carried out on a case-by-case basis. In the case of normality application, the parametric *t*-test was performed for independent samples, and in its absence, the non-parametric Mann–Whitney test was performed to compare the medians.

Multiple linear regression analysis was performed with total job satisfaction score as the dependent variable. Independent variables included WRQoL subscales (Job and Career Satisfaction, General Well-Being, Home–Work Interface, Control at Work, Working Conditions, and Stress at Work) and working sector (school vs clinical). Demographic variables were not included as covariates, as preliminary analyses did not indicate statistically significant associations with the dependent variable. Prior to regression analysis, multicollinearity diagnostics were conducted. Variance Inflation Factor (VIF) values were below recommended thresholds, indicating no multicollinearity concerns among predictors.

For all scales and subscales, the internal consistency of the scales and subscales was calculated by calculating Cronbach’s alpha. The Cronbach’s alpha internal consistency index indicated internal consistency at acceptable levels of the individual subscales and the main scales of the WRQoL and JSS questionnaires, as all calculated values were above the threshold value of 0.60. A multiple linear regression analysis was performed to examine the association between work-related quality of life subscales and working sector with total job satisfaction score as the dependent variable.

### 2.5. Ethics Approval

The study was designed and conducted in accordance with the ethical principles of the Declaration of Helsinki for medical research involving human subjects. All participants provided informed consent and were informed of the voluntary basis of participation and the right to withdraw from the research at any time. Finally, they were informed that their responses would be used exclusively for research purposes, with no individual data disclosed. The research protocol was approved by the Ethical Committee of the Nursing Department (approval reference number: 18/17/10/2024ex, approval date 17 November 2024).

## 3. Results

A total of 165 nurses were included in the study. Most of the participants were female at 77.58% (n = 128) and the vast majority were in the age group 31–40 at 38.18% (n = 63). In terms of educational level, 54.5% of the sample held a postgraduate degree. In terms of work sector between school and clinical nurses during the study period, the relative percentages were almost identical, as for those working as school nurses, the corresponding percentage was 52.1% (n = 86), while for those working as clinical nurses, it was 47.9% (n = 79). In terms of intention to move, clinical nurses expressed a significantly higher desire to move to school nursing (44.3%) compared to school nurses who expressed a desire to return to a clinical setting (17.4%). The distribution of demographic and educational data is presented in Table 1.

Internal consistency reliability was assessed using Cronbach’s alpha coefficients. The Work-Related Quality of Life (WRQoL) scale demonstrated excellent reliability (α = 0.90). Subscale reliability coefficients were α = 0.84 for General Well-Being, α = 0.79 for Home–Work Interface, α = 0.77 for Job and Career Satisfaction, α = 0.69 for Control at Work, α = 0.79 for Working Conditions, and α = 0.69 for Stress at Work.

The Job Satisfaction Survey (JSS) demonstrated high internal consistency (α = 0.87). Subscale Cronbach’s alpha values ranged from α = 0.65 to α = 0.79, indicating acceptable reliability across dimensions. All reliability coefficients exceeded commonly accepted thresholds for internal consistency in behavioral research.

Regarding Job Satisfaction (JSS), the overall mean score was calculated at 125.10 (SD = 22.29), indicating ambivalence or neutrality, that is, moderate levels of job satisfaction among the school and clinical nurses in the study. The highest scores were obtained for the dimensions “Supervision” (M = 17.79) and “Nature of Work” (M = 17.74), while lower scores were obtained for “Additional Benefits” (M = 9.84) and “Salary” (M = 10.68). Regarding WRQoL, the overall mean score was 75.27 (SD = 14.36), indicating a moderate level. Most factors scored low-to-moderate scores, except for the factor “Control at Work” which was at a relatively higher level (M = 10.28) compared to the other variable values, indicating a relatively high level of satisfaction with the quality of professional life, with respect to this factor. (Table 2) The classification of job satisfaction levels was based on established JSS scoring guidelines, where total scores between 108 and 144 indicate moderate (ambivalent) satisfaction. Similarly, WRQoL score interpretation followed instrument-based distributional characteristics and prior validation studies.

### 3.1. Comparison of Job Satisfaction and Quality of Life

Statistical analysis showed that school nurses report significantly higher levels of well-being than clinicians. Specifically, the mean score for the general job satisfaction scale for school nurses was 80.03 (SD = 1.75) compared to 70.09 (SD = 9.76) for clinicians (*p* < 0.001). Similarly, on the job satisfaction scale, school nurses averaged 131.73 (SD = 24.41) versus 117.04 (SD = 17.16) for clinicians (*p* < 0.001). In addition, school nurses scored higher in general well-being, job and career satisfaction, control at work, working conditions, stress at work, and most dimensions of job satisfaction. No statistically significant differences were observed between the two groups in home–work interface, promotion, supervision, fringe benefits, or communication. Detailed comparisons of mean scores, standard deviations, and independent samples *t*-test results are presented in Table 3.

Concerning the effect of demographic characteristics, regression analysis and *t*-test/ANOVA tests showed that in the group of clinical nurses, no factor significantly influenced their job satisfaction and work-related quality of life.

### 3.2. Correlations Between Job Satisfaction and Quality of Life

**Figure 1:**
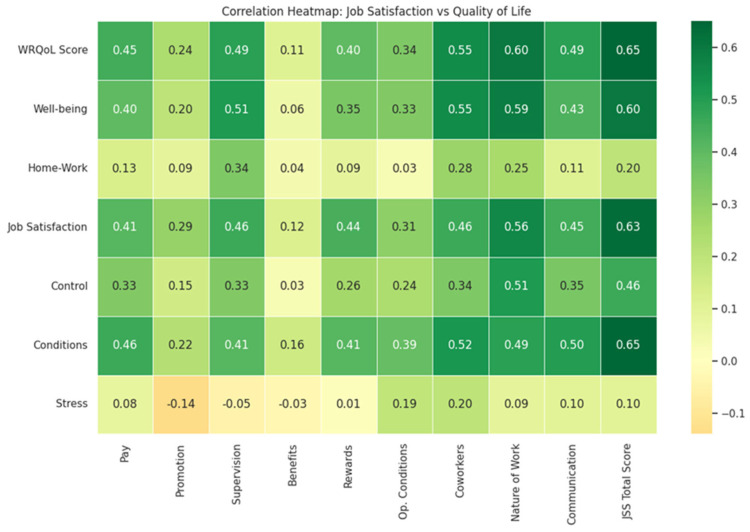
Correlation heatmap between JSS subscales and WRQoL dimensions. Darker green indicates stronger positive correlations (*p* < 0.01).

The relationship between the two research tools was explored through the Spearman coefficient, highlighting strong positive correlations between some variables. The heat map (Figure 1) visualizes the strength of these correlations, highlighting that “Nature of Work” and “Working Conditions” are the variables significantly related to each other in a positive way, whereby an increase in job satisfaction levels equals an increase in work quality of life levels.

The regression model was statistically significant (F = 29.305, *p* < 0.001) and explained 54.7% of the variance in total job satisfaction, with general well-being, job and career satisfaction, and working conditions emerging as significant positive predictors, whereas working sector was not significantly associated with job satisfaction (Table 4).

## 4. Discussion

The current descriptive cross-sectional study aimed to comparatively investigate and determine the level of job satisfaction and Work-related Quality of Life of school and clinical nurses working in public facilities in Greece. In addition, the demographic and work factors influencing these variables were examined, as well as turnover intention, a phenomenon with critical implications for the staffing of the healthcare system. The results of the study showed moderate levels of overall job satisfaction and Work-related Quality of Life for the whole sample. However, benchmarking indicated that school nurses scored statistically significantly higher on both scales compared to their clinical colleagues. The moderate levels of job satisfaction and Work-related Quality of Life in the present study are supported by previous studies in Greece and internationally [12,16,19,23,24,25,26]. Studies conducted in countries such as Norway, Philippines and Spain have shown that the Work-related Quality of Life and work life level of school nurses are high [12,14,17,27].

The statistically significant positive correlation between job satisfaction and work-related quality of life is also documented by previous research [28], and more specifically, an increase in job satisfaction levels equals an increase in occupational/work quality of life levels. The study by Morsy & Sabra (2015) [29] indicated a high, positive, statistically significant correlation between Work-related Quality of Life and job satisfaction among nurses, which is directly related to a reduction in burnout and an increase in job commitment [6]. School nurses reported statistically higher mean scores in job satisfaction and Work-Related Quality of Life compared to clinical nurses. However, these findings should be interpreted with caution given the socio-demographic differences between groups and the use of convenience sampling. School nurses’ job satisfaction may be influenced by factors such as interaction with students and teaching staff, as well as a sense of giving back to the community [29] or experience, age, location and nature of work, level of autonomy and institutional support [12]. Although significant differences were observed at the descriptive level, working sector did not emerge as a statistically significant predictor of job satisfaction in the regression model. Furthermore, no statistically significant differences were observed between participants’ demographic and work characteristics for both clinical and school nurses, which contradicts the studies by Mollas et al., Morsy & Sabra, Gaki et al., and Cimete et al., who identified correlations between the variables [5,19,29,30]. The exception is the gender factor, as female school nurses scored higher mean scores compared to males on both measurement scales, a finding contrary to previous research [31].

The lowest values were found for “salary”, “promotion” and “additional benefits”, while the highest values were found for “supervision”, “nature of work” and the variable “colleagues”. Indeed, job satisfaction is positively influenced by factors such as the nature of the job, supervision and relationships between colleagues, while in contrast, it is negatively influenced by low pay, unfavorable working environment conditions and lack of additional benefits, promotions and recognition [17,30].

On the contrary, the highest average value is for the dimension of “supervision”. These results are consistent with previous studies, which put forward the same factors as determinants for determining the level of job satisfaction [15,17,19,32]. In fact, the group of clinical nurses expresses the greatest dissatisfaction with the dimension “salary”, while school nurses appear most dissatisfied with “additional benefits”. In addition, respondents were asked to indicate optionally whether they would intend to work in a sector other than the one they currently work in. As for school nurses, only 17.44% of the sample stated that they would like to work as a clinical nurse in the future, while the majority at 61.62% gave a negative answer. As for the corresponding case of clinical nurses, 44.3% would like to work as a school nurse and 31.65% faced a dilemma, expressing ambivalence, a finding that could possibly support a trend of mobility or withdrawal from clinical nursing practice. Only 24.06% of all clinical nurses are not available to change their field of work, which may perhaps be justified by the years of work experience, age or job location of clinical nurses. Typically, older nurses express greater job satisfaction while those with higher education are less satisfied [33]. Job satisfaction has been identified as a factor influencing the intention to stay or change job sector and job mobility of nurses [13,34]. Factors related to the work environment rather than individual or demographic factors continue to be the most important for nurses’ intentions to move or leave the profession [35].

Future research should further examine the mechanisms underlying the observed differences between school and clinical nurses, particularly the role of organizational climate, workload, and professional autonomy. Studies incorporating longitudinal designs are needed to clarify the directionality of the relationship between job satisfaction, Work-Related Quality of Life, and turnover intention. Additionally, comparative analyses across different healthcare systems or employment sectors may help identify structural factors influencing nurses’ professional well-being.

### Limitations

This study used convenience sampling, which reduces the representativeness of the sample and therefore the generalizability of the results. The cross-sectional design of the study limits the ability to establish causal relationships between job satisfaction, Work-Related Quality of Life, and associated factors. In addition, research on school nurses is limited, although they play a critical role in providing comprehensive and quality [14,17] health services to the school population, the enhancement of which mandates ensuring a balanced Work-related Quality of Life for school nurses in a conducive and supportive environment to enhance job satisfaction as well.

## 5. Conclusions

The findings of this study indicate moderate overall levels of job satisfaction and Work-Related Quality of Life among nurses. However, statistically significant differences were observed between professional groups, with school nurses reporting higher job satisfaction and Work-Related Quality of Life compared to clinical nurses. Also, the study underscores the need for targeted organizational and policy-level interventions, particularly within the clinical sector, focusing on compensation, career development opportunities, and working conditions. Strengthening these factors may contribute to improved job satisfaction, enhanced quality of work life, and reduced turnover intention among nurses.

## Figures and Tables

**Table 1:** Demographic and professional characteristics of the total sample and comparison between clinical and school nurses.

Characteristics	Total (n = 165)	Clinical Nurses (n = 79)	School Nurses (n = 86)	*p*-Value ^a^
	n (%)	n (%)	n (%)	
Gender				**0.003**
Male	37 (22.4)	26 (32.9)	11 (12.8)	
Female	128 (77.6)	53 (67.1)	75 (87.2)	
Age Group (years)				**0.041**
20–30	50 (30.3)	30 (38.0)	20 (23.3)	
31–40	63 (38.2)	18 (22.8)	45 (52.3)	
41–50	34 (20.6)	17 (21.5)	17 (19.8)	
>50	18 (10.9)	14 (17.7)	4 (4.7)	
Education Level				<**0.001**
BSc (University degree)	70 (42.4)	45 (57.0)	25 (29.1)	
MSc (Master’s degree)	90 (54.5)	32 (40.5)	58 (67.4)	
PhD (Doctoral degree)	5 (3.0)	2 (2.5)	3 (3.5)	
Work Experience				0.082
0–5 years	78 (47.3)	32 (40.5)	46 (53.5)	
6–10 years	38 (23.0)	14 (17.7)	24 (27.9)	
11–15 years	17 (10.3)	9 (11.4)	8 (9.3)	
>15 years	32 (19.4)	24 (30.4)	8 (9.3)	
Employment Status				**<0.001**
Permanent staff	54 (32.7)	40 (50.6)	14 (16.3)	
Contract staff	111 (67.3)	39 (49.4)	72 (83.7)	
Turnover Intention ^b^				**<0.001**
Yes	50 (30.3)	35 (44.3)	15 (17.4)	
No	72 (43.6)	19 (24.1)	53 (61.6)	
Maybe	43 (26.1)	25 (31.6)	18 (20.9)	

^a^ *p*-values derived from Pearson’s Chi-square test. ^b^ Intention to switch to the other nursing setting (Clinical to School or vice versa). Bold values indicate statistical significance (*p* < 0.05).

**Table 2:** Descriptive statistics (Mean, Standard Deviation, Median) of the scales and subscales of Work-Related Quality of Life (WRQoL) and Job Satisfaction (JSS) for the entire sample.

Scale/Subscale	Mean (M)	Standard Deviation (SD)	Median (Md)
WRQoL Scales			
Overall WRQoL (Total Score)	75.27	14.36	79.0
Job and Career Satisfaction (JCS)	20.45	4.58	21.0
General Well-Being (GWB)	20.00	4.94	20.0
Home–Work Interface (HWI)	9.48	2.05	10.0
Control at Work (CAW)	10.28	2.59	10.0
Working Conditions (WCS)	9.73	2.89	10.0
Stress at Work (SAW)	5.33	1.49	5.0
JSS Scales			
JSS Total Satisfaction	125.10	22.29	124.0
Pay	10.68	4.33	10.0
Promotion	10.45	3.38	10.0
Supervision	17.79	4.40	18.0
Fringe Benefits	9.84	3.76	10.0
Contingent Rewards	12.95	4.01	13.0
Operating Conditions	13.28	3.61	13.0
Coworkers	17.21	3.88	18.0
Nature of Work	17.74	4.32	18.0
Communication	15.18	4.06	15.0

**Table 3:** Comparison of Work-Related Quality of Life and Job Satisfaction between Clinical and School Nurses.

	Clinical Nurses Mean (SD)	School Nurses Mean (SD)	t	df	*p*
Overall WRQoL (Total Score)	73.26 (10.3)	83.8 (16.9)	−4.779	163	<0.001
General Well-Being	17.86 (3.4)	21.96 (5.2)	−5.848	163	<0.001
Home–Work Interface	9.26 (1.9)	9.68 (2.1)	−1.316	163	0.190
Job and Career Satisfaction	19.46 (3.4)	21.36 (5.2)	−2.701	163	0.008
Control at Work	9.75 (2.0)	10.75 (2.9)	−2.511	163	0.013
Working Conditions	8.72 (2.2)	10.65 (3.1)	−4.527	163	<0.001
Stress at Work	5.01 (1.2)	5.61 (1.5)	−2.653	163	0.009
JSS Total Satisfaction	117.88 (17.1)	131.73 (24.4)	−4.181	163	<0.001
Pay	9.45 (3.5)	11.80 (4.6)	−3.607	163	<0.001
Promotion	10.55 (3.3)	10.34 (3.4)	0.394	163	0.694
Supervision	17.16 (4.0)	18.36 (4.6)	−1.757	163	0.081
Fringe Benefits	9.92 (3.2)	9.75 (4.2)	0.286	163	0.775
Contingent Rewards	11.82 (3.4)	13.97 (4.2)	−3.570	163	<0.001
Operating Conditions	12.12 (2.9)	14.33 (3.8)	−4.118	163	<0.001
Coworkers	15.72 (2.9)	18.57 (4.1)	−5.043	163	<0.001
Nature of Work	16.22 (3.8)	19.12 (4.3)	−4.563	163	<0.001
Communication	14.88 (3.3)	15.45 (4.6)	−0.897	163	0.371

**Table 4:** Linear regression analysis with total job satisfaction score as the dependent variable and work-related quality of life subscales and working sector as independent variables.

Predictor	B	SE	β	T	*p*	95% CI (Lower–Upper)
Intercept	60.596	8.810	—	6.878	<0.001	43.194–77.998
General well-being	1.141	0.457	0.253	2.495	0.014	0.238–2.044
Home–work interface	−1.315	0.667	−0.121	−1.973	0.050	−2.632–0.001
Job and career satisfaction	1.375	0.551	0.283	2.497	0.014	0.287–2.462
Control at work	−0.248	0.735	−0.029	−0.338	0.736	−1.700–1.204
Working conditions	2.646	0.628	0.343	4.211	<0.001	1.405–3.888
Stress at work	0.335	0.866	0.022	0.387	0.699	−1.375–2.045
School nurses ^a^	2.054	2.714	—	0.757	0.450	−3.307–7.416
Model fit: F = 29.305, *p* < 0.001, adjusted R^2^ = 0.547						

Note. B = unstandardized coefficient; SE = standard error; β = standardized coefficient; CI = confidence interval. ^a^ reference category = Clinical nurses).

## Data Availability

The data presented in this study are available on reasonable request from the corresponding author. The data are not publicly available due to privacy and ethical restrictions, as they contain information that could compromise the confidentiality of research participants.

## Abbreviations

The following abbreviations are used in this manuscript:

WRQoL	Work-Related Quality of Life
JSS	Job Satisfaction Survey
JCS	Job and Career Satisfaction
GWB	General Well-Being
HWI	Home–Work Interface
CAW	Control at Work
WCS	Working Conditions Scale
SAW	Stress at Work
SPSS	Statistical Package for the Social Sciences
SD	Standard Deviation
M	Mean
CI	Confidence Interval
B	Unstandardized Regression Coefficient
SE	Standard Error
β	Standardized Regression Coefficient
ANOVA	Analysis of Variance
